# Disinfectors and Deodorizers

**Published:** 1874-09

**Authors:** 


### DISINFECTORS AND DEODORIZERS.

Infection and ill smells cause a great variety of diseases; they both arise from decomposition and decay of vegetable or animal matter, or both. To deodorize is to take away the bad smell. To disinfect is not only to do this, but to arrest the progress of decay, and thus cut off the supply of a bad odor. Our grandmothers thought they got rid of the ill odor of a sick-room by sprinkling brown sugar on live coals or burning tar; this gave a strong and more agreeable odor; it overpowered the other, so that it was not perceived, but did not destroy it; both odors were really present and the air was doubly impure, only more agreeable to the senses. Hence to deodorize an ill-smelling room or locality intelligently, substances must be used, which, by causing a new chemical combination, destroy the odor altogether; but if the decomposition continues to go on, other odoriferous particles begin to arise requiring a new application of the deodorizer; on this account all deodorizers are efficient only temporarily; hence the only rational method is either to remove the offending material or employ disinfectants, which arrest further decay. If the material both arrests the decay and destroys or absorbs the ill smell, then it is doubly valuable. Two hundred grains of Chloride of Zinc in an ounce of water is a powerful agent in neutralizing bad smells and in arresting both amimal and vegetable decomposition in ships, hospitals, dissecting rooms, cellars, privies, and water closets, without having any ill smell of its own; for disinfecting purposes mix one pint of the above fluid to four gallons of water.

There are three powerful disinfectants: Carbolic Acid, but its smell is objectionable; Chlorine and permanganate of potash; these last two are quite expensive. These disinfectants act by combining with deleterious substances and rendering them harmless, while antiseptics prevent and arrest the decomposition of animal substances.

The only perfect disinfectant is habitual cleanliness and thorough ventilation ; next to that is a dry heat of two hundred and fifty degrees.

The most common and available disinfectant and deodorizer is copperas, crude copperas, sold by druggists at a few cents a pound under the name of sulphate of iron, one pound to two gallons of water to be used as often as necessary to render all odors imperceptible, acting at the same time as an antiseptic, deodorizer and disinfectant, and if instantly thrown over what passes from the body in cholera is one of the cheapest and best means known for preventing its communication to others.

